# Gender Difference in the Association of Metabolic Syndrome and Its Components with Age-Related Cataract: The Korea National Health and Nutrition Examination Survey 2008-2010

**DOI:** 10.1371/journal.pone.0085068

**Published:** 2014-01-08

**Authors:** Young-Hoon Park, Jeong Ah Shin, Kyungdo Han, Hyeon Woo Yim, Won-Chul Lee, Yong-Moon Park

**Affiliations:** 1 Department of Ophthalmology and Visual Science, College of Medicine, The Catholic University of Korea, Seoul, Korea; 2 Department of Biostatistics, College of Medicine, The Catholic University of Korea, Seoul, Korea; 3 Department of Preventive Medicine, College of Medicine, The Catholic University of Korea, Seoul, Korea; 4 Department of Epidemiology and Biostatistics, Arnold School of Public Health, University of South Carolina, Columbia, South Carolina, United States of America; Zhongshan Ophthalmic Center, China

## Abstract

**Purpose:**

To explore the relationship of the metabolic syndrome (MetS) and its components with age-related cataract in a representative Korean population.

**Methods:**

We analyzed the data from the Korea National Health and Nutrition Examination Surveys (2008–2010). A total of 11,076 adults (4,811 men and 6,265 women) aged 40 and over who completed ophthalmologic examination were evaluated. Cataract was defined as the presence of cortical, nuclear, anterior (sub)capsular or posterior subcapsular cataract, from slit-lamp examination or previous cataract surgery. MetS was defined according to the Joint Interim Statement proposed in 2009 from the International Diabetes Federation and the American Heart Association/National Heart, Lung, and Blood Institute.

**Results:**

The prevalence of cataract and MetS in this population was 39.4% (37.1% for men and 41.6% for women) and 38.5% (37.6% for men and 39.4% for women), respectively. Cataract prevalence tended to increase with the number of MetS components in both genders (both P< 0.001). After being controlled for confounders, however, MetS was significantly associated with cataract only in women (adjusted odds ratio (aOR), 1.24; 95% confidence interval (CI), 1.02–1.50]. Reduced HDL cholesterol, elevated fasting glucose, and elevated triglycerides were also significantly associated with cataract in women (aOR, 95% CI; 1.27 (1.07–1.50), 1.23 (1.01–1.50), and 1.26 (1.04–1.52), respectively). In the subgroup analysis for cataract subtype, MetS and reduced HDL cholesterol were significantly associated with nuclear cataract in women (aOR, 95% CI; 1.25 (1.07–1.55) and 1.25 (1.03–1.52), respectively). However, such associations were not found in men.

**Conclusions:**

Our results suggest that MetS and its components appear to be associated with age-related cataract only among Korean women, especially in nuclear cataract.

## Introduction

Cataract is the most common age-related eye disease and a leading cause of blindness and poor vision. The World Health Organization (WHO) reports that cataract is responsible of nearly 50% of blindness in the world (37 million blind people) [1].

In general, the risk factors of cataract are aging, smoking, ultraviolet radiation exposure, and genetic factors [2], [3], whereas the epidemiological evidence is still controversial for antioxidants, alcohol consumption, and supplement use [3]–[6]. Some epidemiological studies have shown positive associations between the risk of cataract and various metabolic abnormalities. Diabetes and hyperglycemia have long been recognized as risk factors for cataract in several studies [7]–[10]. Obesity or central obesity [11]–[15], and serum lipid levels [15]–[17] have also been found to increase the risk of cataract, whereas the relationship between cataract with systolic and diastolic blood pressure is controversial [15], [18], [19].

Recent studies have documented that MetS is associated with ocular disorders such as glaucoma, diabetic and nondiabetic retinopathy [20]–[23]. Although a few previous studies have suggested that MetS is associated with cataract [24]–[28], the association between MetS and its components with cataract has not been properly evaluated in Asian populations because a standardized definition for MetS has not been used in these populations [28].

The present study investigated the association of MetS and its components with age-related cataract and its subtype in a representative Korean population.

## Methods

### Study population

The Korea National Health and Nutrition Examination Survey (KNHANES) is an ongoing cross-sectional survey for the non-institutionalized civilian population of South Korea. A complex, stratified, multistage probability sampling design based on age, sex, and region was used in this survey to represent the Korean population. Since KNHANES IV, a rolling sampling design also has been used so that the samples from each year are independent and homogeneous. KNHANES, coordinated by the Korean Ministry of Health and Welfare, included a health interview survey, a health examination survey, and a nutrition survey. Since 2008, ophthalmologic interviews and examinations have also been conducted. This survey was reviewed and approved by the Institutional Review Board of the Korea Centers for Disease Control and Prevention, and all participants provided written informed consent.

In the present analysis, we limited the study population to adults aged 40 years or older who participated in all three parts of the survey in addition to ophthalmologic interviews and examinations. Therefore, our final study population for the analysis included 11,076 participants (4,811 men and 6,265 women).

### Assessment of cataract

Ophthalmologic examinations were conducted by ophthalmologists from the Korean Ophthalmologic Society in cooperation with the Korea Centers for Disease Control and Prevention (KCDC). Participants underwent a comprehensive eye slit-lamp examination (Haag-Streit BQ-900; Haag-Streit AG, Koeniz, Switzerland) by ophthalmologists. Cataracts were graded according to the Lens Opacities Classification System III (LOCS) standard photographs, regarding nuclear, cortical, anterior (sub) capsular, posterior subcapsular, and mixed type cataract [29], [30]. Pseudophakic and aphakic eyes were included as operated cataracts for the purpose of the statistical analysis. Pseudophakic and aphakic eyes were included as operated cataracts for the purpose of the statistical analysis. However, these phenotypes were not assigned to any subgroup of cataract. In addition, any exclusion criteria for the pseudophakic patients were not applied.

### Measurements

The health interview survey was performed by trained interviewers. All participants were asked about their demographic and socioeconomic characteristics including residential area, education, income, and occupation. The ophthalmologic surveys were also conducted. The subjects were categorized in the group with outdoor activity if they participated in regular outdoor activity for the past ten years. Respondents who were exposed to sunlight more than five hours per day were categorized as the sun exposure group. Family history of eye disease was designated if the subject’s family members in a direct line had any cataract, glaucoma, strabismus, blepharoptosis, retinopathy, or other eye diseases. Respondents were categorized into two groups: ever-smokers (current smokers and ex-smokers) and non-smokers. Alcohol consumption was determined by questioning the participants about their drinking behavior during the month before the health interview. Alcohol consumption status was classified into two groups as non- to moderate drinkers (< 30.0 g alcohol/day) and heavy drinkers (≥ 30.0 g alcohol/day) [31], after converting the average frequency and amount of alcoholic beverages into the amount of pure alcohol (in grams) consumed per day. The subjects who engaged in moderate or vigorous exercise on a regular basis were designated as those who exercised regularly.

Anthropometric measurements of the participants were performed by specially trained examiners. Waist circumference was measured to the nearest 0.1 cm in a horizontal plane at the level of the midpoint between the iliac crest and the costal margin at the end of a normal expiration. The body mass index (BMI) was calculated as the individual’s weight in kilograms divided by the square of the individual’s height in meters. Blood pressure was measured three times on the right arm while the individual was in a seated position after at least 5 minutes of rest using a mercury sphygmomanometer (Baumanometer; Baum, Copiague, NY). The final blood pressure value was obtained by averaging the values of the second and third blood pressure measurements.

Blood samples were obtained after a minimum fasting time of 8 hours. The serum levels of glucose, total cholesterol (TC), high-density lipoprotein (HDL) cholesterol, triglycerides, creatinine, asparate aminotransferase (AST), and alanine aminotransferase (ALT) were measured enzymatically using a Hitachi automatic analyser 7600 (Tokyo, Japan). Hemoglobin (Hb) was measured by the SLS hemoglobin (NoCyanide) method using a XE-2100D (Sysmex/Japan). Insulin resistance was calculated using the homeostasis model assessment (HOMA) estimate of insulin resistance (HOMA-IR  =  fasting insulin [uU/ml] X fasting glucose [mmol/l]/22.5) [32].

### Definition of Metabolic Syndrome

MetS was defined using the criteria proposed by the American Heart Association (AHA) and the National Heart, Lung, and Blood Institute (NHLBI) together with the International Diabetes Federation (IDF) in 2009 [33]. MetS was defined as (1) a waist circumference ≥ 90 cm in men and ≥ 80 cm in women, according to the IDF criteria for Asian countries; (2) a fasting glucose ≥ 100 mg/dl or being on medication use for elevated glucose; (3) fasting triglycerides ≥ 150 mg/dl or cholesterol-lowering medication use; (4) HDL- cholesterol < 40 mg/dL in men and < 50 mg/dL in women or cholesterol-lowering medication use; and (5) systolic blood pressure ≥ 130mmHg and/or diastolic blood pressure ≥ 85 mm Hg or being on an antihypertensive drug treatment for patients with a history of hypertension. MetS diagnosis requires at least three of the five components to be present.

### Statistical analysis

All data are presented as means ± SE for continuous variables or proportions (SE) for categorical variables. Statistical analyses were conducted using the SAS (version 9.2; SAS Institute, Inc., Cary, NC, USA) survey procedure to take into account the complex sampling design with sampling weights of KNHANES and to provide nationally representative prevalence estimates. In order to minimize the effect of variations among the survey years, all the analyses performed in this study were adjusted for survey year. In addition, we conducted stratified analyses to assess the effect modification by gender in the association of MetS and its components with age-related cataract and cataract subtype. Multiple logistic regression analyses were performed to estimate the magnitude of the association of cataract with MetS and its components, and two statistical models based on the characteristics of the variables were used. One model included age and survey year. Subsequently, socioeconomic and lifestyle-related characteristics including income, education, residential area, smoking status, drinking alcohol, exercise, occupation (farmer or fisher), family history of eye disease, and sun exposure were included based on the results from the univariate analysis. The effect modification by gender was also tested by entering the interaction term (gender*MetS) in the full model. A *P* < 0.05 was considered statistically significant.

## Results

### 1. The prevalence and related characteristics of metabolic syndrome

The prevalence of MetS in this population was 38.5% (95% confidence interval (CI), 37.4–39.7%; 37.6% for men and 39.4% for women). The prevalence of individual components of MetS was 40.4% for abdominal obesity, 37.7% for elevated triglycerides, 49.6% for low HDL cholesterol, 45.8% for high BP, and 36.2% for elevated glucose. Table 1 shows the characteristics of the participants by MetS status. Subjects with MetS were more likely to be older in both genders. Urban residence, higher education, and family history of eye disease were lower, whereas low income, occupation of farmer or fisher, sun exposure, and regular exercise were higher in women with MetS. Occupation of farmer or fisher and regular exercise were lower, whereas heavy drinker was higher in men with MetS.

**Table 1:** Characteristics of the participants according to the presence or absence of metabolic syndrome by gender.

	Overallproportion	Total		*P*	Men		*P*	Women		*P*
		absence	presence		absence	presence		absence	presence	
Sex (%)				0.134						
male	48.5(0.4)	49.8(0.7)	47.9(0.9)							
female	51.5(0.4)	50.2(0.7)	52.1(0.9)							
Age (years)				<.001			<.001			<.001
40–49	38.4(0.8)	56.8(1.1)	10.0(0.9)		45.0(1.3)	34.5(1.5)		49.9(1.2)	17.8(1.1)	
50–59	29.2(0.7)	32.4(0.9)	24.4(1.0)		30.0(1.1)	33.0(1.4)		28.5(0.9)	29.0(1.2)	
60–69	17.5(0.5)	8.9(0.5)	30.6(0.8)		14.8(0.7)	20.8(1.0)		12.5(0.7)	27.0(1.0)	
≥70	14.9(0.5)	1.8(0.2)	35.0(1.2)		10.2(0.6)	11.7(0.8)		9.0(0.6)	26.2(1.2)	
Residence (urban)	74.9(2.1)	76.2(2.0)	73.7(2.2)	0.013	74.6(2.3)	76.1(2.3)	0.348	77.8(2.0)	71.5(2.4)	<.001
Education (>6yrs)	67.0(0.8)	74.6(0.9)	58.4(1.2)	<.001	78.9(1.0)	79.5(1.2)	0.699	70.2(1.1)	39.1(1.4)	<.001
Income (lowest quartile)	21.6(0.7)	17.2(0.8)	26.4(1.0)	<.001	16.8(0.9)	18.1(1.0)	0.289	17.7(0.9)	34.0(1.3)	<.001
Occupation (farmer/fisher)	10.2(1.1)	10.3(1.2)	10.1(1.1)	0.833	13.3(1.6)	10.2(1.3)	0.010	7.3(0.9)	10.0(1.2)	0.003
Outdoor activities (yes)	98.3(0.4)	98.4(0.4)	98.3(0.5)	0.794	98.8(0.3)	98.1(0.6)	0.084	97.9(0.6)	98.5(0.6)	0.295
Sun exposure (yes)	25.7(1.0)	25.1(1.1)	26.6(1.3)	0.179	33.6(1.4)	32.8(1.5)	0.660	16.7(1.1)	20.8(1.6)	<.001
Family history of eye disease (yes)	18.1(0.5)	20.6(0.7)	15.6(0.7)	<.001	18.2(0.9)	17.1(1.2)	0.471	22.9(0.9)	14.2(0.9)	<.001
Ever-smoker (yes)	44.1(0.5)	44.1(0.8)	44.6(1.0)	<.001	81.1(0.9)	83.7(1.1)	0.074	7.3(0.6)	8.7(0.7)	0.136
Heavy drinker (yes)	18.0(0.6)	16.2(0.7)	20.7(1.0)	<.001	24.1(1.1)	30.8(1.5)	<.001	6.1(0.7)	5.4(0.8)	0.505
Regular exercise (yes)	25.5(0.7)	27.4(0.8)	24.0(0.8)	<.001	29.1(1.1)	25.4(1.3)	0.017	74.3(1.0)	77.3(1.2)	0.025

Data are presented as the means±SE or % (SE).

### 2. The prevalence and related characteristics of cataract

**Figure 1:**
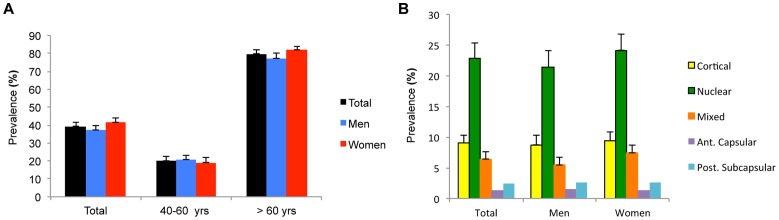
Prevalence of age-related cataract according to gender: by age group (A) and by the subtype of cataract (B). The error bars represent the upper 95% confidence intervals.

The prevalence of cataract in participants aged 40 years or older was 39.4% (95% CI, 37.2–41.6%; 37.1% for men and 41.6% for women) and that in participants aged 60 years or older over was 79.8% (95% CI, 77.5 – 82.1%; 77.2% for men and 81.8% for women). The prevalence was 22.8% (95% CI, 20.3 – 25.3%) for nuclear type, 9.1% (95% CI, 7.8 – 10.4%) for cortical type, 4.2% (95% CI, 2.9 – 8.7%) for mixed type, 0.7% (95% CI, 0.3 – 1.3%) for anterior(sub)capsular type, and 0.3% (95% CI, 0.0 – 0.7%) for posterior subcapsular type in participants aged 40 years or older (figure 1). Table 2 shows characteristics of the participants by age-related cataract. These results indicate that prevalence of cataract tended to increase with age in both genders (*P*< 0.001). The prevalence of cataract was higher in women than men (*P*<0.001). Urban residence, higher education, family history of eye disease, and heavy drinker were lower, whereas low income, occupation of farmer or fisher, and sun exposure were higher in both genders with cataract. Ever-smoker was higher in men, and regular exercise was lower in women.

**Table 2:** Characteristics of the participants according to the presence or absence of age-related cataract by gender.

	Overallproportion	Total		*P*	Men		*P*	Women		*P*
		absence	presence		absence	presence		absence	presence	
Sex (%)				<.001						
male	48.5(0.4)	50.4(0.7)	45.7(0.7)							
female	51.5(0.4)	49.6(0.7)	54.3(0.7)							
Age (yrs)				<.001			<.001			<.001
40–49	38.4(0.8)	56.8(1.1)	10.0(0.9)		57.9(1.3)	11.6(1.1)		55.8(1.3)	8.7(1.0)	
50–59	29.2(0.7)	32.4(0.9)	24.4(1.0)		31.7(1.1)	28.4(1.5)		33.1(1.1)	20.9(1.0)	
60–69	17.5(0.5)	8.9(0.5)	30.6(0.8)		8.7(0.6)	31.6(1.3)		9.2(0.6)	29.8(1,0)	
≥70	14.9(0.5)	1.8(0.2)	35.0(1.2)		1.8(0.3)	28.4(1.3)		1.9(0.3)	40.6(1.4)	
Residence (urban)	74.9(2.1)	79.2(2.0)	68.3(2.8)	<.001	78.4(2.1)	69.7(2.9)	<.001	80.1(2,0)	67.1(2.8)	<.001
Education (> 6 yrs)	67.0(0.8)	81.5(0.8)	44.5(1.2)	<.001	87.0(0.9)	63.1(1.5)	<.001	75.8(1.0)	28.8(1.4)	<.001
Income (Lowest quartile)	21.6(0.7)	12.5(0.7)	35.8(1.2)	<.001	10.9(0.7)	30.0(1.3)	<.001	14.1(0.8)	40.8(1.4)	<.001
Occupation (farmer/fisher)	10.0(1.1)	6.9(0.8)	14.8(1.8)	<.001	8.5(1.0)	17.2(2.2)	<.001	5.2(0.7)	12.7(1.6)	<.001
Outdoor activities (yes)	98.3(0.4)	98.6(0.4)	97.9(0.7)	0.207	99.0(0.4)	98.0(0.7)	0.194	98.3(0.4)	97.9(0.7)	0.405
Sun exposure (yes)	25.7(1.0)	21.7(0.9)	32.0(1.7)	<.001	29.9(1.2)	39.0(1.8)	<.001	13.3(0.9)	26.1(1.9)	<.001
Family history of eye disease (yes)	18.1(0.5)	21.5(0.7)	12.9(0.7)	<.001	20.5(1.0)	12.5(0.9)	<.001	22.5(0.9)	13.2(0.8)	<.001
Ever-smoker (yes)	44.1(0.5)	44.9(0.7)	42.8(0.8)	0.048	81.7(0.8)	82.7(1.0)	<.001	7.5(0.6)	9.2(0.7)	0.078
Heavy drinker (yes)	18.0(0.6)	19.6(0.7)	14.8(0.9)	<.001	28.9(1.1)	22.8(1.3)	<.001	7.4(0.7)	3.4(0.6)	<.001
Regular exercise (yes)	25.5(0.7)	27.0(0.8)	23.3(1.0)	0.002	28.1(1.1)	25.7(1.4)	0.161	25.8(0.9)	21.3(1.1)	<.001

Data are presented as the means±SE or % (SE).

### 3. The associations of metabolic syndrome with age-related cataract

**Figure 2:**
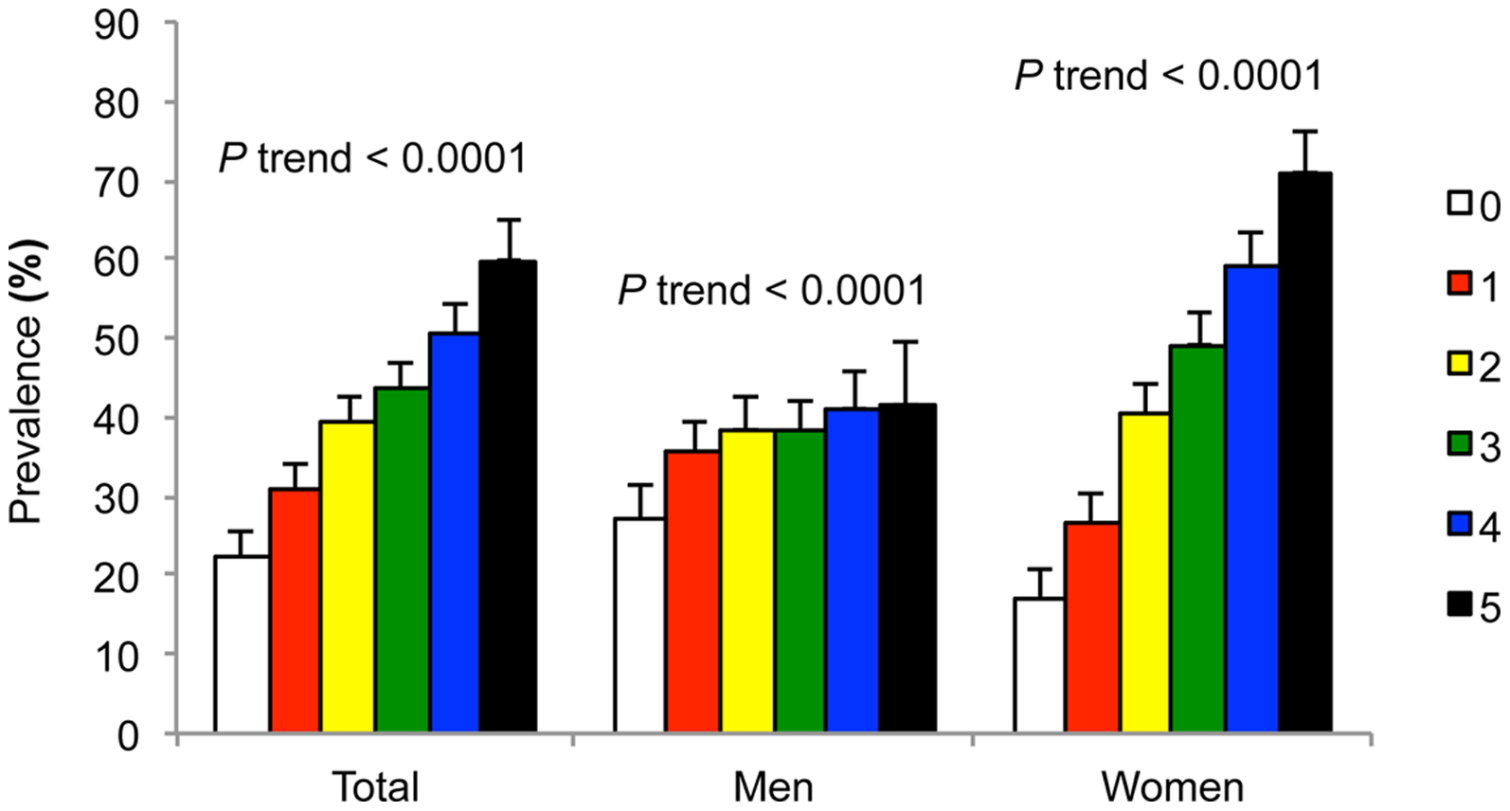
Prevalence of age-related cataract according to the increasing number of MetS components by gender. The error bars represent the upper 95% confidence intervals. All *P* for trend were < 0.0001.

**Figure 3:**
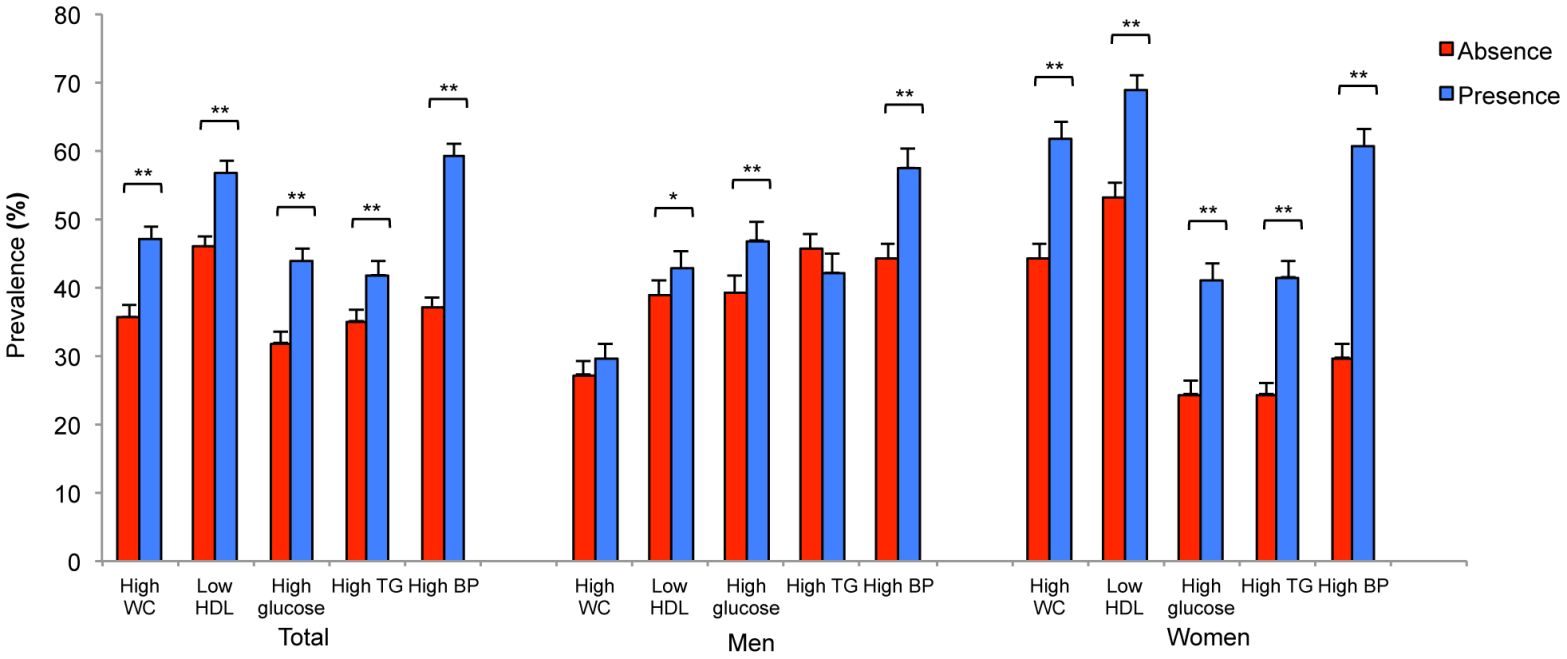
Prevalence of age-related cataract according to the absence or presence of MetS components by gender. The error bars represent the upper 95% confidence intervals (*P<0.05, **P<0.0001).

Figure 2 shows that the prevalence of cataract rose with an increase in the number of metabolic syndrome components in both genders (both *P* <0.0001), although the increasing trend was larger in women. Figure 3 shows the gender difference in the relationship between prevalence of cataract and metabolic syndrome components. The prevalence of cataract was higher in all MetS components for women (all *P* <0.0001), but it was higher only in low HDL (*P* = 0.031), high glucose (*P* <0.0001), and high BP (*P* <0.0001) for men.

Table 3 shows the results of multiple logistic regression analyses. There were significant effect modifications of gender on age (*P*  = 0.023) in the full model, which supported the rationale for stratified analyses by gender. After being controlled for confounders, MetS was significantly associated with cataract in women (adjusted odds ratio (aOR), 1.24; 95% confidence interval (CI), 1.03–1.49). Cataract prevalence increased with the number of MetS components in women (P =  0.007). Especially, the odds of cataract in subjects with 4 to 5 MetS components were significantly higher compared with subjects with none to one MetS component (aOR, 1.47; 95% CI, 1.12–1.93). Reduced HDL cholesterol, elevated fasting glucose, and elevated triglycerides were also significantly associated with cataract in women (aOR, 95% CI; 1.26 (1.07–1.49), 1.23 (1.02–1.49), and 1.26 (1.04–1.52), respectively). In the subgroup analysis for cataract subtype, MetS and reduced HDL cholesterol were significantly associated with nuclear subtype in women (aOR, 1.24; 95% CI, 1.01–1.54 and 1.24 (1.02–1.50), respectively). Also, the prevalence of the nuclear subtype of cataract increased with the number of MetS components in women (*P* =  0.037).

**Table 3:** Association of metabolic syndrome and its components with age-related cataract by gender.

Variables	Total cataract				Cortical subtype				Nuclear subtype			
	Model1		Model2		Model1		Model2		Model1		Model2	
	OR(95% CI)	*P*	OR(95% CI)	*P*	OR(95% CI)	*P*	OR(95% CI)	*P*	OR(95% CI)	*P*	OR(95% CI)	*P*
Male												
MetS (presence vs. absence)	1.00(0.83,1.20)	0.973	0.95(0.79,1.14)	0.548	0.93(0.67,1.29)	0.646	0.90(0.63,1.26)	0.543	1.13(0.89,1.43)	0.331	1.07(0.85,1.34)	0.572
Number of MetS components												
0–1	1		1		1		1		1		1	
2	1.15(0.90,1.46)		1.11(0.87,1.43)		1.19(0.84,1.70)		1.16(0.82,1.63)		1.08(0.78,1.48)		1.04(0.74,1.44)	
3	1.11(0.87,1.40)		1.05(0.82,1.34)		0.89(0.58,1.36)		0.86(0.55,1.34)		1.25(0.92,1.70)		1.17(0.86,1.60)	
4–5	1.00(0.77,1.30)		0.92(0.71,1.20)		1.10(0.71,1.72)		1.06(0.67,1.67)		1.06(0.74,1.52)		0.99(0.71,1.39)	
P for trend	0.885		0.634		0.93		0.907		0.463		0.767	
MetS component												
High waist circumference	1.02(0.85,1.23)	0.830	0.95(0.80,1.15)	0.615	0.83(0.58,1.18)	0.288	0.84(0.59,1.21)	0.323	1.23(0.97,1.55)	0.082	1.14(0.91,1.42)	0.264
Low HDL	0.99(0.83,1.19)	0.944	0.01(0.84,1.22)	0.888	0.90(0.66,1.22)	0.485	0.93(0.68,1.27)	0.791	1.06(0.83,1.34)	0.659	1.09(0.85,1.39)	0.519
High glucose	1.03(0.86,1.22)	0.757	1.01(0.85,1.20)	0.949	0.94(0.69,1.28)	0.685	0.90(0.66,1.24)	0.521	1.14(0.91,1.43)	0.245	1.14(0.92,1.41)	0.248
High triglycerides	1.00(0.83,1.19)	0.963	0.95(0.80,1.14)	0.611	1.19(0.87,1.62)	0.278	1.11(0.82,1.52)	0.543	0.97(0.78,1.22)	0.817	0.95(0.76,1.18)	0.637
High blood pressure	1.04(0.86,1.26)	0.692	1.00(0.82,1.22)	0.990	1.27(0.93,1.72)	0.133	1.24(0.92,1.69)	0.180	0.92(0.71,1.18)	0.511	0.87(0.68,1.12)	0.278
Female												
MetS (presence vs. absence)	1.21(1.01,1.46)	0.036	1.24(1.03,1.49)	0.030	0.84(0.62,1.14)	0.274	0.89(0.66,1.19)	0.430	1.23(1.01,1.50)	0.048	1.24(1.01,1.54)	0.046
Number of MetS components												
0–1	1		1		1		1		1		1	
2	1.19(0.93,1.53)		1.23(0.94,1.61)		0.85(0.56,1.30)		0.91(0.59,1.40)		1.17(0.85,1.59)		1.20(0.86,1.68)	
3	1.24(0.99,1.55)		1.25(0.98,1.60)		0.62(0.43,0.91)		0.67(0.46,0.96)		1.33(1.01,1.75)		1.34(1.01,1.80)	
4–5	1.39(1.08,1.80)		1.47(1.12,1.93)		0.94(0.61,1.44)		1.03(0.67,1.58)		1.30(0.96,1.76)		1.37(0.99,1.90)	
P for trend	0.011		0.007		0.503		0.797		0.046		0.037	
MetS component												
High waist circumference	1.09(0.90,1.32)	0.388	1.11(0.91,1.36)	0.297	0.75(0.55,1.00)	0.051	0.78(0.57,1.06)	0.115	1.12(0.88,1.41)	0.364	1.15(0.90,1.48)	0.270
Low HDL	1.27(1.08,1.50)	0.005	1.26(1.07,1.49)	0.006	1.15(0.87,1.52)	0.318	1.14(0.87,1.50)	0.336	1.26(1.04,1.52)	0.018	1.24(1.02,1.50)	0.031
High glucose	1.19(0.98,1.43)	0.065	1.23(1.02,1.49)	0.030	0.98(0.72,1.34)	0.903	1.01(0.74,1.39)	0.952	1.13(0.90,1.41)	0.294	1.20(0.95,1.51)	0.132
High triglycerides	1.20(0.99,1.44)	0.052	1.26(1.04,1.52)	0.020	1.03(0.76,1.39)	0.842	1.12(0.84,1.50)	0.442	1.14(0.92,1.40)	0.236	1.18(0.95,1.46)	0.140
High blood pressure	1.03(0.86,1.24)	0.755	1.08(0.89,1.30)	0.463	0.99(0.74,1.31)	0.932	1.07(0.80,1.42)	0.614	1.00(0.81,1.24)	0.992	1.03(0.83,1.29)	0.772

Data are presented as the odds ratio (95% confidence interval).Abbreviation: MetS, Metabolic syndrome; HDL, high density lipoprotein cholesterol.

Model 1: adjusted by age and survey year; model 2: adjusted by socioeconomic and lifestyle-related characteristics including income, education, residential area, smoking status, drinking alcohol, exercise, occupation (farmer or fisher), family history of eye disease, and sun exposure.

However, no significant association between MetS and cataract was found in men. In addition, there was no association between MetS and other subtypes of cataract including anterior (sub)capsular, posterior subcapsular, and mixed type cataract.

## Discussion

To the best of our knowledge, this is the first large population based study to examine the association of age-related cataract with MetS and its components among a representative Korean population stratified by gender. In this cross-sectional study, conducted as part of KNHANES 2008–2010, MetS and its components including reduced HDL cholesterol, elevated fasting glucose, and elevated triglycerides were positively associated with the risk of age-related cataract only in women. In addition, the prevalence of age-related cataract increased with the number of MetS components in women. These phenomena were also observed in the nuclear subtype of cataract.

Gender difference in the association between MetS and age-related cataract was consistent with the data from some existing studies [16], [17], [24], [25], [27]. In the present study, there was no significant relation between MetS and cataract in Korean men. The reason is not clear but a few studies have reported strong associations of some components of MetS with cataract among women [15], [17], [24]. This gender specific association might be explained by differences of hormonal [34] and life-style related characteristics [35] of MetS between men and women. In addition, there has been some evidence that gender difference in MetS contributes to the gender-related differential risk of cardiovascular disease [36].

MetS components could be potential risk factors of cataract, especially as all of them are associated with age. The data about the relationship between cataract and all five MetS components are controversial in many studies [10], [13], [16], [18], [20], [37]. Galeone et al. [27] and Sabanayagam C et al. [28] reported that cataract prevalence increased with increasing number of metabolic syndrome components in both men and women. However, Lindblad BE et al. [25] suggested that MetS and its components, abdominal adiposity, diabetes, and hypertension, seem to be associated with an increased risk for cataract extraction, especially among women aged less than 65 years.

Our study found a tendency for an increase in the percent rate of cataract among women with reduced HDL cholesterol. Similar data were found in the Beaver Dam Eye Study: higher serum HDL cholesterol was associated with a decreased risk of cataract [17]. A strong association was found between low levels of HDL cholesterol and the development of lens opacities of adults of both genders from South Africa [38]. Animal studies have shown acceleration of the development of diabetic cataracts by hyperlipidemia and low HDL in rats [39]. Inflammation and oxidative stress resulting from reduced HDL cholesterol levels could induce cataract formation [28], [40]–[42]. These findings are in contrast to the results of Sabanayagam C et al. [28]. They reported that there were no significant associations between high-serum triglycerides or low-serum HDL and cataract in a Malay population. However, an association was observed between low HDL levels and cortical cataract in their study. In addition, some studies have reported associations of high-triglyceride of low-HDL levels with cataract among specific subpopulations as in our report [16], [24], [38]. Especially, Paunksnis A et al. [24] have suggested that the percent rate of cataract was significantly higher among women with higher arterial pressure, central obesity, and elevated triglyceride level than among women without those MetS components. The results of these studies correspond well with the present study.

High glucose has been considered a risk factor for cataract in several epidemiologic studies, both cohort and case-control studies [7], [9], [15], [43]. No significant association was observed in a cohort study [24]. Diabetes may be related to cataract by glycation of the lens proteins [44], [45]. Diabetes has been shown to be associated with cataract in several Asian populations [28], [46]. Mechanisms proposed to explain increased cataractogenesis from elevated blood glucose and diabetes include non-enzymatic glycosylation of lens proteins, leading to oxidation, cross-linking, aggregation, and precipitation, and polyol accumulation, in which aldose reductase catalyzes the conversion of glucose to sorbitol [26], [47]. Sorbitol accumulation generates increased osmotic pressure, causing swelling and eventual rupture of lens fiber cells [48]. Lens proteins are highly susceptible to the advanced glycation events because of their continuous exposure to the elevated environmental glucose levels [49].

Many studies have found a positive association of cataract with high blood pressure, especially systolic hypertension in both genders [18], [24], [37]. In the Blue Mountains Eye Study, hypertension was associated with a lower prevalence of nuclear cataract [50]. In the POLA Study, hypertension decreased the risk of cataract surgery [43], while in a study from Italy, hypertension was associated with an increased risk of cataract extraction in women [15]. However, conflicting findings on the relationship between blood pressure and cataract exist across other studies [7], [18], [19], [26], [37], [50], [51]. In the present study, there was no association between elevated blood pressure and cataract in both Korean men and women. On balance, current evidence suggests that neither hypertension nor antihypertensive medications are likely to be major cataract risk factors.

In the subgroup analysis for cataract subtype, MetS and reduced HDL cholesterol were significantly associated with nuclear cataract in women (aOR, 95% CI; 1.24 (1.01–1.54) and 1.24 (1.02–1.50), respectively). However, no significant association between MetS and cataract was found in men. These findings are different from the Singapore Malay Eye Study in that MetS showed a modest association with cortical subtypes, although this association might be biased due to not using a standard definition of MetS. [28].

The relationship between cataract subtypes and MetS components is controversial in the previous studies. Klein BE et al. [17] suggested that higher HDL cholesterol was associated with decreased risk of cortical cataract in women. Jacques PF et al. [13] reported that diabetes and measures of adiposity were unrelated to the prevalence of cortical and nuclear cataract. The Framingham Studies findings suggested that fasting hypertriglyceridemia ≥ 250 mg/dL was associated with the increased risk of posterior subcapsular cataract in men but no associations were noted between serum lipid/lipoprotein variables and risk of cortical or nuclear cataract [16]. We could not suggest the possible explanation for the association of MetS and its components with nuclear cataract in detail. However, Tan JSL et al. reported that the presence of MetS was associated with an increased risk of incident cortical, posterior capsular cataract and nuclear cataract, and baseline diabetes predicted nuclear cataract after considering age and other factors in The Blue Mountains Eye Study. [26] Although Tan JSL et al’s and our findings are generally inconsistent with previous findings for the association of MetS with cortical or posterior subcapsular cataract, the reason for the disparity of findings may also partly be due to racial/ethnic differences in the study population and use of different grading systems for cataract and lens opacities. Further studies are warranted to confirm the relationship between MetS and nuclear cataract.

The prevalence of cataract in adults Koreans aged 40 years or older was 38.5% (37.6% for men and 39.4% for women). The prevalence of cataract was similar to those reported in other Asian populations [28], [29], where no statistically significant differences in prevalence were observed in relation to gender. However, women showed a significantly higher prevalence of cataract than men in the present study.

Our study should be interpreted with consideration of the following limitations. First, this study was a cross-sectional analysis; therefore, causal relationships could not be identified, nor were the mechanisms of these associations explored. Second, cataracts were graded according to the Lens Opacities Classification System III (LOCS) standard photographs, regarding only nuclear, cortical, anterior (sub)capsular, posterior subcapsular, and mixed type cataract but the grading of cataract was not measured. Therefore, the relationships cannot be assessed between the severity of cataract and MetS components in this study.

Despite these limitations, this study used a nationally representative sample of adults in Korea, which is a crucial strength of our study. Also, cataract was diagnosed by direct slit-lamp examination. Moreover, to the best of our knowledge this is the first large population-based study in Asia to examine the association between MetS based on the strict definition and age-related cataract.

Taken together, MetS was significantly associated with age-related cataract in women. In addition, reduced HDL cholesterol, elevated fasting glucose, and elevated triglycerides were significantly associated with cataract in women. In the subgroup analysis for cataract subtype, MetS and reduced HDL cholesterol were significantly associated with nuclear cataract in women. However, such associations were not found in men. In conclusion, our results suggest that there may be gender differences in the association between MetS and age-related cataract in Korean adults.
